# Synchrony and Physiological Arousal Increase Cohesion and Cooperation in Large Naturalistic Groups

**DOI:** 10.1038/s41598-017-18023-4

**Published:** 2018-01-09

**Authors:** Joshua Conrad Jackson, Jonathan Jong, David Bilkey, Harvey Whitehouse, Stefanie Zollmann, Craig McNaughton, Jamin Halberstadt

**Affiliations:** 1 Department of Psychology and Neuroscience, University of North Carolina, Chapel Hill, USA; 2 Institute for Evolutionary and Cognitive Anthropology, University of Oxford, Oxford, UK; 3 Research Centre for Psychology, Behaviour and Achievement, Coventry University, Coventry, UK; 4 Department of Psychology, University of Otago, Dunedin, New Zealand; 5 Animation Research Limited, Dunedin, New Zealand

## Abstract

Separate research streams have identified synchrony and arousal as two factors that might contribute to the effects of human rituals on social cohesion and cooperation. But no research has manipulated these variables in the field to investigate their causal – and potentially interactive – effects on prosocial behaviour. Across four experimental sessions involving large samples of strangers, we manipulated the synchronous and physiologically arousing affordances of a group marching task within a sports stadium. We observed participants’ subsequent movement, grouping, and cooperation via a camera hidden in the stadium’s roof. Synchrony and arousal both showed main effects, predicting larger groups, tighter clustering, and more cooperative behaviour in a free-rider dilemma. Synchrony and arousal also interacted on measures of clustering and cooperation such that synchrony only encouraged closer clustering—and encouraged greater cooperation—when paired with physiological arousal. The research helps us understand why synchrony and arousal often co-occur in rituals around the world. It also represents the first use of real-time spatial tracking as a precise and naturalistic method of simulating collective rituals.

## Introduction

Social theorists have long argued that collective rituals—that is, rituals performed in groups—function to build or enhance social cohesion^1–8^. This idea is supported by extensive ethnography, which describes in detail how such rituals foster relational ties in pre- and post-agricultural societies^9,10^. More recently, social scientists have also begun to contribute to this evidence base by testing specific causal hypotheses via experimental methods. Much of this work has focused on two salient aspects of collective rituals: synchronised behaviour and shared physiological arousal. These studies have provided tests of synchrony and arousal’s independent causal effects on prosocial behaviour, but have seldom examined their interaction. Moreover, they have often been limited to dyads or small groups, even though rituals in the real world often involve much larger groups of participants^11^. Here, we investigate how synchrony and arousal affect group cohesion and cooperation in large naturalistic groups using a newly developed real-time tracking paradigm, hoping to provide novel insights into the mechanisms of ritual’s dramatic effects.

## Synchrony, Arousal, and Prosociality

Behavioural synchrony—the matching of rhythmic behaviour between individuals—is a recurring feature of collective rituals around the world^12^. Dancing, singing, drumming, chanting, and marching are all common examples of synchronised behaviours that feature in collective rituals. Recent experimental evidence finds that diverse forms of synchrony serve to increase prosocial attitudes and behaviour (e.g., cooperation, compassion, helpfulness, liking) towards ritual participants and third parties^13–17^, at least in the absence of intergroup conflict. Shared physiological arousal, including pain, is another (less common) feature of rituals, from college fraternity initiations to Hindu *kavadi* rituals of self-mutilation, which also appears to promote social cohesion and cooperation both in the field^18^ and in the laboratory^19^.

These two distinctive features of rituals have been studied independently in the laboratory, but the mechanisms underlying them remain unclear. Some research^20,21^ has suggested that synchrony increases auditory or visual attention to other ritual participants, which fosters shared goals and social bonds^22^. Other studies have relied on Durkheim’s notion of “collective effervescence” —the process by which an energising group activity increases positive affect and social cohesion—to explain synchrony’s effect on prosocial behaviour^23^. Hove^24^ and others^25^, in contrast, have argued for an “identity fusion” account, in which synchronous behaviour blurs the lines between the self and others, creating a sense of oneness within a group. An overlapping set of mechanisms have been proposed to underlie physiological arousal. Konvalinka and colleagues^26^, for example, found evidence for the role of collective effervescence among walkers and spectators in a fire-walking ritual, while Whitehouse and Lanman^8^ argued that self-focused attention during arousal consolidates existing bonds among fused group members (see also^27,28^). These mechanisms are not mutually exclusive, of course, and may function differently in different contexts.

Despite their co-occurrence in many real rituals, from military drills, to Afro-Brazilian drumming, to Maasai *adumu* dancing, synchrony and physiological arousal have rarely been studied in the same paradigm. However, an examination of their interaction can provide insights into their mechanistic relationship. Specifically, if the two factors are underpinned by the same mechanism (e.g., collective effervescence), combining them in a ritual should produce a negative interaction, such that synchrony will have smaller effect in the context of arousing ritual, and arousal a smaller effect in the context of a synchronous one. In contrast, if they operate via distinct mechanisms, their interaction should be neutral or positive, such that the effect of one factor is independent or possibility facilitative of the other.

Only two very recent studies have experimentally manipulated synchrony and physiological arousal simultaneously and neither found interactive effects. The first measured cooperation among rowers who wore headphones playing a beat that varied in its speed and in its synchronicity with others rowers^29^. The researchers found a main effect of rowing speed on subsequent cooperation, but no main or interactive effects of synchrony. It is unclear whether this is due to their use of a specialist sample of competitive rowers or, as they suggest, the limitations of their synchrony manipulation, which limited participants’ ability to coordinate^22,30^. The second study measured self-reported closeness among triads of high school students who performed synchronised or independent movements to music, with high or low “exertion” (i.e., while standing or sitting;^31^). Participants felt closer to their other two group members when they performed the same (versus different) physical movements, and when their movements required more exertion. There was a trend toward a positive interaction (*p* = 0.13), such that the combination of synchrony and exertion produced more closeness than either alone. These results, based on self-reports, are suggestive, but behavioural measures are needed to ensure that the findings do not reflect participants’ causal theories or desires rather than (or in addition to) their perception of closeness^32,33^.

Equally important, these few laboratory studies have examined only small groups. Larger samples are needed not only to mimic the conditions in many real-world rituals, but also to gain further insight into how those rituals work, as the mechanisms discussed above make competing predictions when implemented on larger scales. For example, whereas attention and coordination are more difficult in larger groups compared to smaller ones, collective effervescence is potentially more energising in larger groups compared to smaller ones. Meanwhile, a self-activation account would predict no effect in large groups of strangers, who are unlikely to be included in one another’s self-concept. Therefore, studying synchrony and arousal in large, naturalistic groups (versus the small laboratory groups that have typified research on the subject) not only provides greater ecological validity, but also insight into the psychological mechanisms underlying ritual’s effects on social behaviour.

## The Present Study

The present study built on the strengths of previous efforts by manipulating both synchrony and physiological arousal in a marching paradigm that allowed for auditory and visual coordination, and measured groups’ affiliative and cooperative behaviour unobtrusively in a naturalistic setting^34^. To simulate conditions found in actual marching rituals (e.g., military groups), we ran our study on large groups (*N*s = 39–47), which required the use of a larger venue than a traditional psychology laboratory: a fully enclosed professional sports stadium in Dunedin, New Zealand. We observed group behaviour by tracking participants’ movements using a high-definition camera (of which participants were unaware) mounted 25 m directly above them (i.e., “*in vivo* behavioural tracking”; see^35–37^ for more methodological detail).

We measured social cohesion and cooperation via three behavioural tasks. First, we administered a “group formation task,” during which participants were instructed to organise themselves, over a series of nine trials, into groups. Participants’ desire to affiliate with others in this task was operationalised in terms of the size of the groups they joined, consistent with social ecologists’ treatment of prosociality and group size^38^.

Second, participants were given the opposite goal, a “dispersal task” in which they first clustered around a central point before being asked to break away from the group and to find a new place in the experimental venue to stand. Cohesion in this task was operationalised as the average distance between each individual and the rest of the participants after dispersing—where lower distance represented greater cohesion. Physical proximity has a long history in psychology as a measure of interpersonal closeness and bonding, from the classic literature on “personal space”^39^ and proxemics^40,41^, to correlational field studies linking physical and psychological closeness^42^, to modern computer simulations of group formation^43^. These and many other studies show that people do not approach each other arbitrarily, and that physical distance maps closely onto self-reported attraction^44^, relational commitment^45^, and other affiliative cues^46^.

Finally, we administered a “cooperation task”^47^, in which participants had to work together to pick up 500 small washers scattered across the experimental area. This task resembled a classic N-person cooperation dilemma, described by Archeti and Sheruing (^48^, p. 10) as “a collective action problem for the production of a common good.” In our specific task, picking up washers represented the common good—since participants could not complete the experiment until they had picked up all washers and deposited them in a small basin—whereas the temptation to free ride and rely on others’ efforts represented the collective action problem. Just as in other free-rider problems (e.g. voting, blood donation), it was in both the individual’s and group’s interest to collect the washers, but the contribution of any particular individual was both negligible and virtually undetectable, creating an incentive to save energy by doing nothing and letting other participants do the work. As in previous social loafing paradigms (see^49^), cooperation was operationalized by participants’ efforts—represented in this task by their mean search speed—to overcome this temptation.

Our hypotheses were that synchrony and arousal would each facilitate larger groups, less dispersal, and greater effort in the cooperation task. We made no a priori prediction regarding the interaction between synchrony and arousal, given the dearth of previous research.

## Method

### Participants

One hundred and seventy-two individuals (*M*_age_ = 21.43, *SD* = 4.50, range = 17–41; 41 men, 130 women, 1 who identified as “other”) were recruited in Dunedin, New Zealand, through a student employment website. A chi-squared test confirmed that gender ratios did not vary across the four experimental sessions, X^2^ (6, *n* = 172) = 3.93, *p* = 0.69. Sessions did vary based on the average age of participants, *F* (3, 168) = 3.08, *p* = 0.03, such that the “no synchrony, low-arousal” session had significantly younger participants than the “synchrony, low-arousal” session (*M*_diff_ = 2.65, *Tukey HSD p* = 0.03). However, controlling for age in all analyses did not substantively change our results, indicating that age was not a confounding variable. Table 1 contains further information about each session.

**Table 1:** Characteristics of Each Experimental Session.

Session	*n*	Ages	Genders
Synchrony, low arousal	47	22.96	11 men, 36 women
No synchrony, low arousal	39	20.31	8 men, 31 women
Synchrony, arousal	45	20.84	13 men, 31 women, 1 other
No synchrony, arousal	41	21.39	9 men, 32 women

Participants were explicitly instructed not to sign up with friends, and participants who indicated knowing another individual in their session were reassigned prior to participation. Participants were paid NZ$30 to compensate them for travel costs. All participants gave written, informed consent before participation and were fully debriefed after completing the study. They were also given the option (which nobody took up) to have their video data deleted from the sample.

### Venue

The study was conducted at the Forsyth-Barr Stadium, Dunedin. An Elphel NC535 network camera was mounted 25 m overhead, and continuously captured video on the 30 m × 20 m experimental area for the duration of the study, at 30 frames/second at the full resolution of 2592 × 1944 pixels. The Theia SY110 lens used provided a 120° view with almost 0% distortion. Following data collection, individual participants were tracked using custom software developed by Animation Research Ltd. (See the Halberstadt and colleagues^35^ supplemental materials for more detail.)

### Procedure

After indicating their availability by email, participants were randomly assigned to one of 4 conditions, representing a fully crossed 2 (synchronous versus asynchronous movement) × 2 (high versus low physiological arousal) design. This random assignment was critical, because it minimized the risk of systematic variance across sessions that was not related to our manipulations and would have created problematic nestedness in our data.

**Figure 1:**
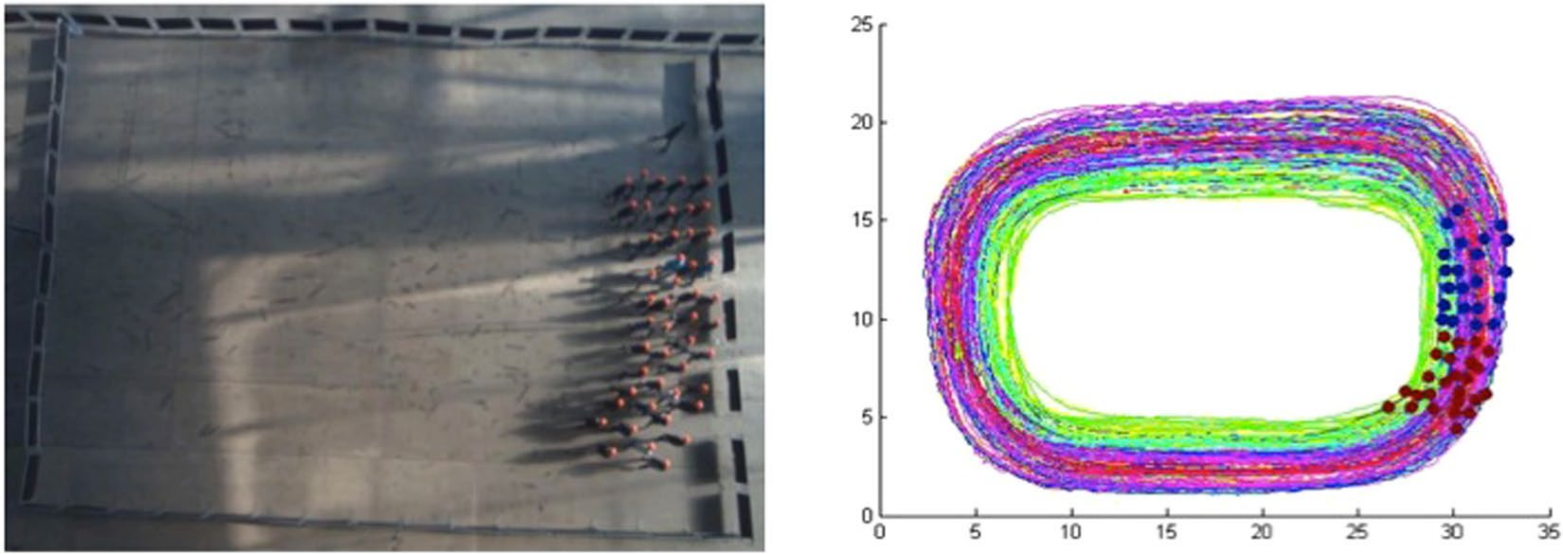
Experimental marching task (synchrony condition) as seen from the hidden camera (left panel) and as reconstructed by our tracking software (right panel). Coloured lines in the right panel correspond to participants’ movements over the tracking period.

As their first task, all participants were asked to follow a research assistant, at his pace, around the perimeter of the experimental area for five minutes (timed by the experimenter). Participants in the synchrony condition, but not the control condition, were instructed to walk in step with the experimenter and with their peers. Participants were instructed to walk in rank formation so that they could more easily synchronise with the participants to their sides and in front of them. In addition, the groups were independently assigned (by experimental session) to one of the two physiological arousal conditions, differentiated only by the speed at which the research assistant walked. In the high arousal condition, the research assistant walked approximately 50% faster (2.0 m/s) than average pedestrian walking pace (1.4 m/s;^50^), while in the low arousal condition, the research assistant walked slightly below the average walking speed (1.2 m/s). See Fig. 1 for an illustration of this marching task.

After completing the marching task, participants took part in three tasks to assess social cohesion, which we term “group formation,” “dispersal,” and “cooperation,” respectively. In the group formation task, participants were asked to stand around the perimeter of the experimental area in the order of their randomly assigned participant numbers. Participants started in this position so that there would be no differences in clustering across conditions before they formed groups. Participants were then asked to take five steps forward—into the centre of the area—and to “form groups of any size and composition,” signalling their success by remaining stationary and raising their hands. The experimenter then directed participants to form “new groups” starting from their current location, and then to repeat the task a third time. The whole set of three group formations (beginning with assembly around the perimeter) was then repeated two more times, for a total of nine trials.

In the dispersal task, participants were instructed to assemble close together in the center of the space, and then to “find another place to stand in the experimental area.” Thus, participants had the choice of moving in whatever direction, and however far, they wished.

**Figure 2:**
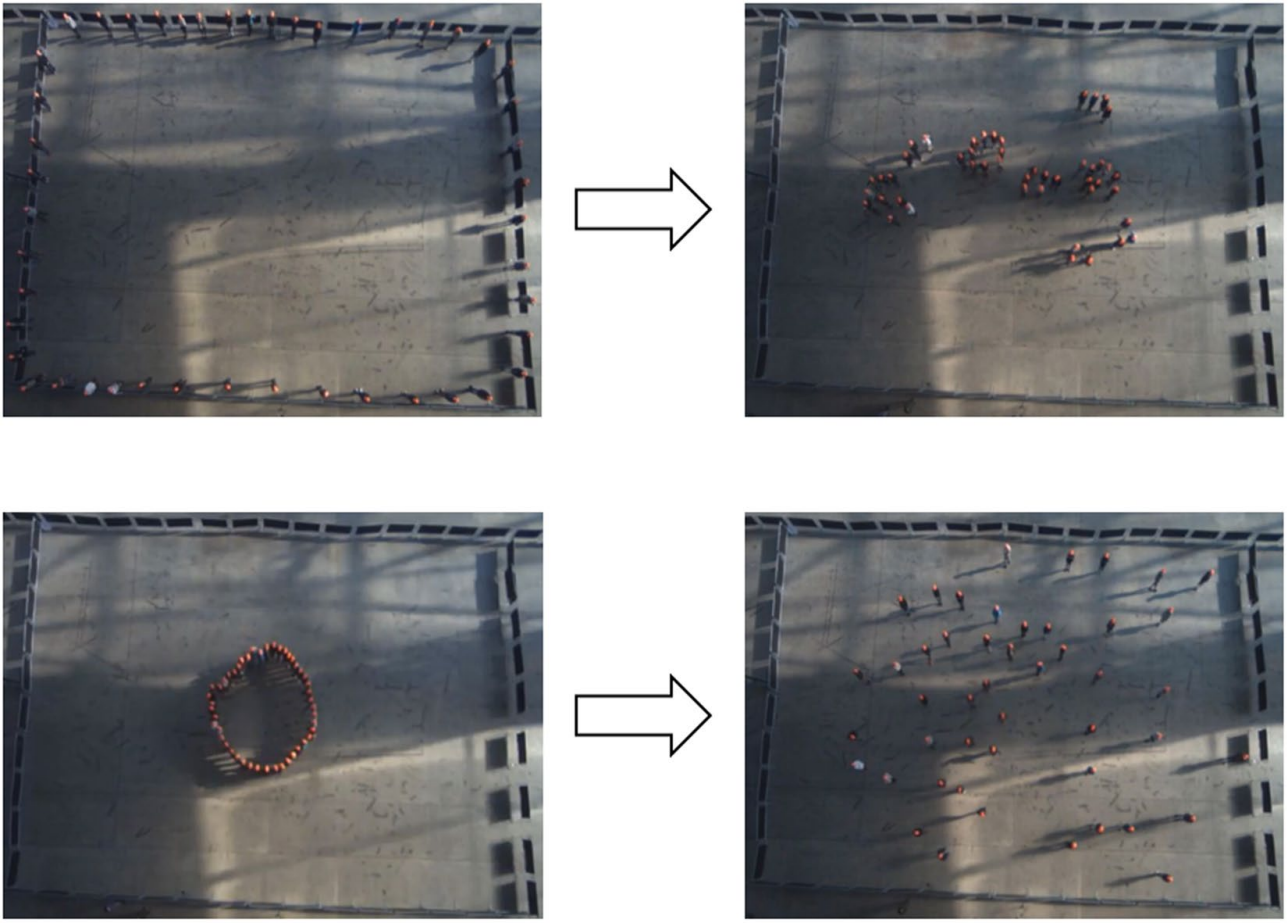
Group formation (top panels) and dispersal (bottom panels). The left-hand panels depict participants at the beginning of the tasks. The right-hand panels depict participants at the conclusion of the tasks.

Finally, in the cooperation task, participants were instructed to collect 500 1-inch washers, which had been scattered across the experimental area prior to their arrival, and to deposit them, one at a time, in a bin located in a corner of the experimental space. Participants were told that the experiment would not end until each of the washers had been found and deposited (a research assistant monitored the bin and determined when the task was complete). See Fig. 2 for images captured during the group formation and dispersal tasks.

### Measurement and Data Analysis

Our tracking software produced a series of x-y coordinates for each participant throughout the experiment. A custom MATLAB script used these coordinates to derive analysable parameters (e.g. interpersonal distance and speed). This script also used a k-means cluster analysis, supplemented by a silhouette procedure that optimised the ratio of within-cluster to between-cluster distance to determine subgroups during the grouping phase of the study. All data are available at https://osf.io/u4fg7/.

### Ethics

This study was approved by the ethics committee at the University of Otago. All participants provided written informed consent to participate in the study, and all methods were performed in accordance with relevant guidelines and regulations.

## Results

As expected, group sizes in the group formation task and dispersal distance (the average distance between a participant and all other participants at the end of the dispersal task) were significantly correlated with each other (*r* = −0.28, *p* < 0.001), and with walking speed in the cooperation task (*rs* = 0.55 and *r* = −0.38, *ps* < 0.001): participants who joined larger groups also tended to remain close to others in the dispersal task, and to put more effort into finding the washers. We find this correlation to be particularly important, suggesting that an underlying factor accounted for significant variance across our three constructs. Gender and age were not significantly related to any of the metrics of cohesion or cooperation (*p*s > 0.20). Finally, readers should bear in mind that participants in our analyses were aware of one another’s behaviour, since they interacted in collectives. Since we randomly assigned participants to their experimental session, this artefact does not violate the statistical assumptions of our models, but readers should nevertheless interpret small effects in this study with caution.

### Group Formation

Across all sessions, participants formed a total of 227 groups. Group sizes were normally distributed, with a mean size of 6.8 members (*SD* = 3.0), and a median and mode of 6. To examine the effects of synchrony and physiological arousal on group size, we constructed a repeated measures multilevel model predicting group size, with observations nested within trials (*n* = 9) and participants (*n* = 172) in a cross-classified design^51^. Synchrony, physiological arousal, and their interaction term were entered at participant-level predictors. Intercepts were modelled as varying across participants to account for the nested data structure. Parameters were estimated using a restricted maximum likelihood algorithm.

In this model, participants in the synchrony condition formed significantly larger groups than those in the control condition, *b* = 0.19, *SE* = 0.08, *t* = 2.05, *p* = 0.04, and participants in the high-arousal condition formed significantly larger groups than those in the low-arousal condition, *b* = 0.68, *SE* = 0.08, *t* = 8.28, *p* < 0.001. The synchrony × arousal interaction did not reach significance, *b* = 0.12, *SE* = 0.08, *t* = 1.43, *p* = 0.15. These results replicated in a model controlling for group dispersal in each trial (e.g. how spread out participants stood as they formed groups), with synchrony, *b* = 0.27, *SE* = 0.08, *t* = 3.35, *p* < 0.001, and arousal, *b* = 0.57, *SE* = 0.08, *t* = 7.12, *p* < 0.001, once more facilitating larger grouping, and no significant interaction, *b* = 0.05, *SE* = 0.08, *t* = 0.69, *p* = 0.49. This suggested that synchrony and arousal were not simply encouraging larger grouping because participants were clustering together during group formation.

### Dispersal

**Figure 3:**
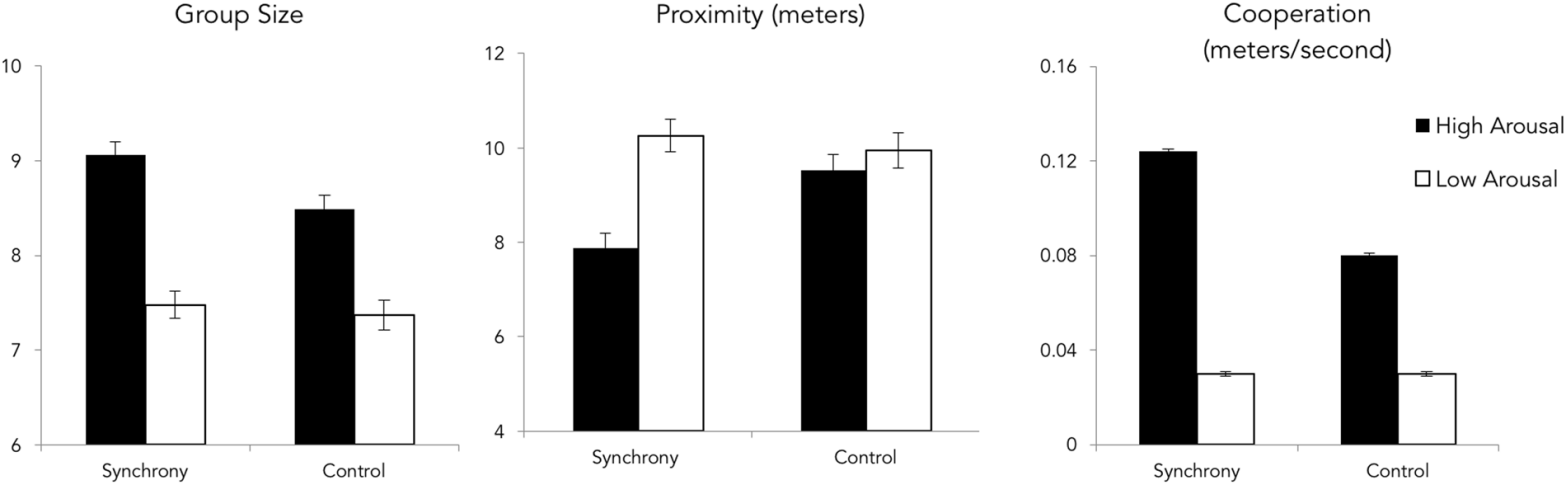
Group size (far left), interpersonal proximity during dispersal (middle) and speed during the cooperation task (far right) as a function of the arousal and synchrony conditions.

At the end of the dispersal task, participants stood an average of 9.39 m (*SD* = 2.33) from others in the study, with a median distance of 8.92 m. A 2 × 2 ANOVA revealed main effects of both synchrony, *F*(1,171) = 4.04, *p* = 0.046, *d* = 0.31, and physiological arousal, *F*(1,171) = 18.00, *p* < 0.001, *d* = 0.66. Participants who marched in synchrony (*M*s = 9.08 versus 9.74, *SE*s = 0.23 versus 0.24), and those who marched quickly (*M*s = 8.71 versus 10.11, *SE*s = 0.23), subsequently stood closer to each other, compared to participants with no synchrony instructions and slow walkers, respectively. There was also a significant interaction between synchrony and physiological arousal (see Fig. 3), *F*(1,171) = 8.87, *p* = 0.003, *d* = 0.46. Under conditions of low arousal, synchrony did not have a significant effect on proximity (*M*s = 10.26 versus 9.94, *SE*s = 0.32 versus 0.35), *t*(1, 84) = 0.69, *p* = 0.49, *d* = 0.14, but under high physiological arousal, synchrony significantly increased proximity (*M*s = 7.88 versus 9.52, *SE*s = 0.32 versus 0.34), *t*(1, 84) = 3.51, *p* = 0.001, *d* = 0.75. See Fig. 3 for a depiction of these effects.

### Cooperation

During the foraging task, participants moved an average of 0.07 m/s (*SD* = 0.04), with a median speed of 0.06 m/s and a range from 0.02 m/s to 0.14 m/s. A 2 × 2 ANOVA revealed main effects of synchrony *F*(1,171) = 698.91, *p* < 0.001, *d* = 4.13, and physiological arousal *F*(1,171) = 2546.97, *p* < 0.001, *d* = 7.52, such that both synchronous (*M*s = 0.09 versus 0.06, *SE*s = 0.001 versus 0.001) and aroused (*M*s = 0.10 versus 0.04, *SE*s = 0.001) participants walked faster during the foraging task, compared to controls. There was also a significant interaction of synchrony and physiological arousal on foraging speed, *F*(1,171) = 60.48, *p* < 0.001, *d* = 1.21. Under conditions of high physiological arousal, the effect of synchrony on foraging speed produced a greater mean difference (*M*s = 0.12 versus 0.08, *SE*s = 0.0005), *t*(1, 84) = 18.12, *p* < 0.001, *d* = 3.94, than under conditions of low physiological arousal (*M*s = 0.05 versus 0.03, *SE*s = 0.001), *t*(1, 84) = 28.59, *p* < 0.001, *d* = 6.28. See Fig. 3 for illustration of these effects.

An alternative explanation for the effects of physiological arousal on cooperation is that participants in the high arousal condition were simply primed to walk quickly by their initial marching task. To explore this alternative, we tested participants’ walking speed during the dispersal task, which also featured uninterrupted walking. A synchrony × arousal ANOVA on dispersal speed revealed no main effects of physiological arousal, *F*(1, 171) = 2.07, *p* = 0.15, *d* = 0.22, or synchrony, *F*(1, 171) = 0.05, *p* = 0.83, *d* < 0.0001, and no interaction, *F*(1, 171) = 2.15, *p* = 0.14, *d* = 0.23, and controlling for participants’ dispersal speed did not affect the aforementioned effects on cooperation (*ps* < 0.001, *d*s > 1.00). These results suggest that differences in cooperation were not an artefact of the physiological arousal manipulation, or merely manifestations of general differences in participant speed throughout the experiment.

## Discussion

This study conceptually replicates previous findings that behavioural synchrony^12–17^ and shared physiological arousal^18–20^ in small groups independently increase social cohesion and cooperation. We also identify several novel effects regarding the relationship between synchrony and physiological arousal. For instance, we find that, across all dependent variables, arousal exerted a stronger effect than synchrony, and that synchrony only influenced interpersonal distance—and influenced effort in the cooperative task to a greater extent—when accompanied by high physiological arousal. Our results were clearest for group cooperation in our free-rider dilemma, which is arguably the most face valid of our dependent measures. However, the relative similarity of our effects across multiple dependent variables provides convergent support for the effects of synchrony and arousal on prosociality in large groups.

Although both factors increased prosociality, effects were reliably stronger for arousal than for synchrony, a fact that, when considered in light of previous research, hints at distinct mechanisms. For instance, studies have documented a critical role of auditory and visual coordination in synchrony—but not physiological arousal—such that individuals’ synchronisation must be reinforced via sensory information (e.g. music) in order to produce cohesion^20–22^. Indeed, the published experimental research on synchrony and cooperation has been limited to dyads and small groups, where it is relatively easy to visually and audibly coordinate with group members. In this study’s marching task, where participants could only see their immediate neighbours, synchrony’s sensory reinforcement may have been lacking, which could have disrupted its cohesive properties. While this account of our findings seems plausible, it should be taken with caution—and considered alongside direct analyses of synchrony’s mechanisms^52^—since this experiment did not include direct process measures.

Of more significance, synchrony and arousal interacted in a positive way on two of our three measures, such that physiological arousal boosted synchrony’s prosocial effects (the interaction on group size was consistent with this pattern but not statistically significant), a pattern inconsistent with redundant mechanisms. One possibility that has been foreshadowed by previous studies is that large-scale synchrony—where coordination is difficult—only produces behavioural cohesion when group members have a high awareness of their identity. According to evidence reviewed by Whitehouse and Lanman^8^, physiological arousal directs attention towards people’s self and social identity. In the context of large groups, then, behavioural synchrony might only translate to intergroup cohesion and cooperation when arousal has rendered identity concerns salient. The possibility is supported by recently published research, which has found that large chanting rituals—which entail both physiological arousal and synchrony—increased affiliation in groups of 20–30 students^53^. However, since that study did not independently manipulate synchrony and arousal, future research is needed to test the account directly. A simple alternative account, for example, is that participants’ footsteps were louder and more frequent in our high-arousal condition, providing more salient target for attention.

We note that we have operationalized “arousal” in the current study in terms of physical exertion. Although our approach is consistent with other recent research on identity fusion^27^, it is not the only way to conceptualize the construct. Other investigations, for example, have found that psychological arousal associated with highly memorable dysphoric events (e.g. wars) increases group bondedness^28^. Psychological and physiological arousal are sometimes subjectively interchangeable, and may be causally related, but they are conceptually distinct. Indeed, in their theoretical discussion of arousal, Whitehouse and Lanman^8^ distinguish these two forms of arousal, and argue that “pure” physiological arousal should increase cohesion in groups that are already bonded to a certain extent, whereas dysphoric events should create fusion bonds amongst strangers. Our results do not directly contradict this framework, in that arousal’s effects on cohesion were strongest when paired with synchrony, which independently bonded groups together. However, we also observed strong independent effects of arousal on cooperation, even in the absence of synchrony. This raises the possibilities that participants in our synchrony-control condition felt a degree of social bond that arousal increased, perhaps because they had already completed several activities as a group.

Another possibility is that shared arousal has independent properties that increased cohesion amongst strangers. Classic literature on the misattribution of arousal, for example, suggests that adrenergic arousal (via adrenaline administration) can be mistaken for other emotions, even love^54^. At the very least, our results point to multiple paths by which physiological arousal can increase cohesion, which should be explored and teased apart by future research. For example, future studies could explicitly explore how individual versus shared arousal contribute to prosociality. It is possible that physiological arousal is only socially bonding when individuals go through an arousing event together. Experimentally induced physiological arousal in individuals may not produce this effect, and may even elicit aggression under some conditions (e.g. when paired with confrontation)^55^. In a similar vein, future research should test whether shared physiological arousal and synchrony will increase or decrease cohesion with members from an *out-group*. Previous research has found that synchrony can increase compliance with instructions to aggress against out-groups^56^, and increase the perceived ease of intergroup aggression^34^. However, other studies have shown that synchrony can increase self-reported prosociality towards out-group members and non-performers^57^. Future research is needed to clarify how synchrony shapes perceptions of out-group individuals, and how these effects differ on the basis of whether they are co-performers in the synchronous activity, among many other factors.

One limitation of the present research was our reliance on four experimental sessions, each which contained a unique experimental condition. This limitation was necessary in order to test for synchrony and arousal’s effects in larger groups. However, it also meant that participants in our experiment were to some extent interdependent. In order to minimize any problematic data nestedness, we randomly assigned participants to their experimental sessions, and held all characteristics constant across sessions that were not related to our manipulations of synchrony and physiological arousal. Nevertheless, readers should interpret our effects with caution, especially the small main effect of synchrony during group formation and dispersal. Moreover, we encourage future studies to replicate our effects with multiple groups that can be tested in random effect models.

Although motivated by an interest in collective rituals, we acknowledge that, in the present study, we did not manipulate one of rituals’ defining characteristics: causal or functional opacity^58,59^. In cognitive terms, rituals—in contrast to instrumental behaviour—are “socially stipulated group conventions opaque from the perspective of physical causality” (^60^, p42; see also^61–63^). Future research should therefore directly manipulate causal opacity in order to measure how it interacts with synchrony, arousal, and other common elements of collective rituals. Over the past decade, social scientists have successfully fractioned ritualistic behaviour into these individual elements; we believe that researchers should now begin to recombine these elements in order to fully examine rituals as they naturally occur.
